# Sleeping Beauty: Anesthesia May Promote Relapse in Dogs With Diffuse Large B-Cell Lymphoma in Complete Remission After Chemo-Immunotherapy

**DOI:** 10.3389/fvets.2021.760603

**Published:** 2021-11-22

**Authors:** Eugenio Faroni, Silvia Sabattini, Jacopo Lenzi, Dina Guerra, Stefano Comazzi, Luca Aresu, Alessia Mazzanti, Stefano Zanardi, Veronica Cola, Emilio Lotito, Laura Marconato

**Affiliations:** ^1^Department of Veterinary Medical Sciences, Alma Mater Studiorum University of Bologna, Bologna, Italy; ^2^Department of Biomedical and Neuromotor Sciences, Alma Mater Studiorum University of Bologna, Bologna, Italy; ^3^Department of Veterinary Medicine, University of Milano, Lodi, Italy; ^4^Department of Veterinary Sciences, University of Torino, Turin, Italy; ^5^Clinica Veterinaria Dell'Orologio, Sasso Marconi, Bologna, Italy

**Keywords:** anesthesia, dog, immunosuppression, lymphoma, propofol, TIVA, isoflurane

## Abstract

Surgery-induced stress and anesthesia-related immunosuppression are believed to play a critical role in human oncology patients. Studies have hypothesized that anesthesia influences patients' outcome, promoting tumor recurrence and metastasis. Aim of the study was to investigate whether anesthesia promoted relapse in dogs with diffuse large B-cell lymphoma (DLBCL). Medical records were searched for dogs with DLBCL, that were in complete remission (CR) after the same chemo-immunotherapy protocol. Dogs receiving anesthesia were included if the procedure was performed while in CR. Time to relapse (TTR) was obtained via Kaplan–Meier method. Association between anesthesia and relapse was assessed using a nested case-control design and estimated using conditional logistic regression. Sixty-one dogs with DLBCL were included. Overall median TTR was 329 days (95% CI, 281–377). Forty-eight (79%) dogs relapsed during the study period, while 13 (21%) were still in CR at data analysis closure. Eighteen (30%) dogs received anesthesia with opioids, propofol, and isoflurane or sevoflurane. The relative risk of lymphoma relapse for dogs undergoing anesthesia was significantly higher compared with dogs not undergoing anesthesia, with an odds ratio of 3.09 (*P* = 0.019) on multivariable analysis. Anesthesia may promote relapse in dogs with DLBCL treated with chemo-immunotherapy, although a role of perioperative stress cannot be ultimately excluded. Considering the high frequency of anesthetic procedures required for diagnostic and therapeutic protocols among oncology patients, it is of utmost interest to characterize the effects of single anesthetic agents on the immune system. Further prospective studies are needed to better define the impact of anesthesia on patients' outcome.

## Introduction

The link between the immune system and cancer has been widely appreciated but also debated for over a century, due to the ability of the immune responses to mediate protection against cancer, but also to promote cancer progression (1, 2).

There are several ways by which immunity can be impaired including, among others, surgery-induced stress responses and anesthesia-induced immunosuppression promoted by the stimulation of the hypothalamic-pituitary-adrenal axis and the sympathetic nervous system (3, 4). As a result, pre-existing quiescent micrometastasis may become active following surgery, thereby favoring neoplastic progression (3).

Studies have suggested that volatile anesthetics and opioids may contribute to the suppression of cell-mediated immunity (3–6). Conversely, propofol-based total intravenous (TIVA) and regional anesthesia may have protective effects by reducing the need for opioids or volatile anesthetics, or reducing the stress response to surgery (6–8).

In the clinical setting, whether the anesthetic choice impacts cancer patient outcome, therefore suggesting a procedural change, is a contentious issue and a matter of debate.

The first study referring to a possible association between cancer, immune system, and anesthesia was published in 1977, and reported a higher survival rate in human breast cancer patients receiving anesthesia with halothane during surgery, compared to those receiving ether (9). More recently, few retrospective studies reported an improved outcome for human oncology patients undergoing surgery with propofol anesthesia compared to volatile drugs (10–12). These findings represent the basis for most of the ongoing prospective clinical trials (e.g., NCT01975064, NCT03034096, NCT02786329, and NCT02840227).

In dogs, a transient immunosuppressive effect of anesthesia has also been demonstrated, promoting depression of T-cell blastogenesis and a decrease in NK cytotoxic function (13, 14). Nevertheless, to the authors' knowledge, a possible *in vivo* interaction between canine cancer and anesthesia has never been documented. Dogs with cancer often need anesthesia for invasive diagnostic and/or therapeutic procedures. Additionally, better treatment modalities have prolonged survival in veterinary oncology patients; as a result, the likelihood that these same patients will undergo anesthetic procedures for cancer-unrelated reasons throughout their lives has also increased.

Diffuse large B-cell lymphoma (DLBCL) is the most common lymphoma histotype both in humans and dogs, accounting for nearly half of the diagnosed cases (15, 16). Although CHOP (cyclophosphamide, doxorubicin, vincristine, and prednisolone) or CHOP-based protocols are considered the gold-standard, recent advances in veterinary medicine have led to the development of chemo-immunotherapy protocols, resulting in improved outcomes (17, 18). Yet, the majority of dogs are not cured and will eventually die for their disease. Relapses not only are more difficult to treat, but also demotivate owners, who often elect euthanasia once lymphoma recurs.

The aim of this retrospective study was to investigate whether anesthesia promoted relapse in dogs with DLBCL that were in complete remission after treatment.

## Materials and Methods

Medical records were retrospectively searched for dogs with completely staged DLBCL that received the same chemo-immunotherapy protocol and were in complete remission (CR) based on a negative minimal residual disease assessed by flow cytometry and/or PCR for antigen receptor rearrangements (PARR) at the end of treatment.

For all dogs, initial staging consisted of history and physical examination, complete blood count with differential, serum biochemistry profile, thoracic radiographs, abdominal ultrasound, cytologic evaluation of liver and spleen regardless of the ultrasonographic appearance, and immunophenotype determined by flow cytometry (FC) on a lymph node aspirate, peripheral blood (PB) and bone marrow (BM) aspirate. In all dogs, lymphadenectomy of a peripheral enlarged lymph node was performed to obtain lymphoma histotype, according to WHO guidelines (19).

With prior written informed owner consent, all dogs were treated with a CHOP-based protocol with the incorporation of APAVAC immunotherapy, as previously described (17).

End-staging was carried out at the end of treatment, and every clinical, radiological, ultrasonographic or laboratory investigation that disclosed abnormalities at pre-treatment staging was repeated. PB and BM were sampled again for FC analysis or PARR in all cases, regardless of the initial degree of infiltration. After completion of chemo-immunotherapy, a regular monthly follow-up was required for the first year, and every 2 months thereafter.

For the purpose of the current study, only dogs achieving CR and having a follow-up of at least 270 days were included in the analysis. Because dogs with DLBCL receiving chemo-immunotherapy have a quite long median time to progression (> 300 days) (17, 18), we opted for a minimum follow-up time slightly longer than those commonly applied to veterinary medicine (i.e., 270 days from the obtainment of complete response). This was arbitrarily selected in order to ensure an adequate time interval for the monitoring of patients and the measurement of exposure. Dogs receiving anesthesia were included only if the procedure was performed while in CR. For these dogs, the reason for the procedure and anesthetic drugs were retrieved.

Relapse was defined as the clinical reappearance and cytologic evidence of lymphoma in any anatomic site in dogs having experienced CR, and time to relapse (TTR) was defined as the time interval between the obtainment of clinical complete response and relapse.

### Statistical Analysis

Categorical variables were summarized as frequency (percentage), while numerical variables were summarized as median (range). Non-normality of numerical data was assessed using the Shapiro–Wilk test. TTR was obtained with the Kaplan–Meier method and expressed as median [95% confidence interval (CI)]. Dogs lost to follow-up or those still in CR at the end of the study were censored for TTR analysis.

Cox proportional hazards regression analysis was performed to assess the possible association of the following variables with TTR: anemia (yes vs. no), thrombocytopenia (yes vs. no), serum lactate dehydrogenase (LDH) activity (increased vs. normal), WHO stage (stage V vs. stage I–IV), substage (b vs. a), BM infiltration by means of FC (yes vs. no), extranodal site involvement (yes vs. no), and pre-treatment with steroids (yes vs. no). According to laboratory reference ranges, cutoffs for anemia, thrombocytopenia, and increased LDH activity were set at hematocrit <37%, total platelet number <160,000/mm^3^, and serum LDH activity >130 IU/l, respectively. The cutoff for BM infiltration was set at >3% of CD45^+^ cells, as it has been shown to negatively affect outcome in dogs with DLBCL (20). Site of extranodal involvement, when present, was registered.

Association between anesthesia (exposure) and relapse (outcome) was assessed using a nested case-control design. Dogs experiencing relapse were defined as cases, and 2 controls were randomly selected and matched to each case by follow-up duration. Dogs were considered exposed if surgery requiring anesthesia was detected prior to the matching date. We chose this approach to ensure an equal time window for measuring time-dependent exposure in cases and controls (21). An illustrative example of this technique is provided in Supplementary Figure 1. The association between exposure and outcome was estimated using conditional logistic regression, which is appropriate for a time-matched nested case-control study. Results were expressed as odds ratios that, for the incidence density sampling used in this study, provide unbiased estimates of the relative risks (rate ratios) in the underlying cohort. The two groups, obtained via incidence density sampling, were compared by means of Fisher's exact test to detect statistically different distribution of the prognostic variables listed above. Thus, regression models included as additional covariate the propensity score of getting anesthesia, given the subset of baseline factors that showed to be differently distributed across the two exposure groups with a significance level of *P* ≤ 0.20.

All analyses were carried out by using Stata software, version 15 (StataCorp. 2017. Stata Statistical Software: Release 15. College Station, TX: StataCorp LP), and IBM SPSS Statistics for Windows, version 25.0 (Armonk, NY, USA). The significance level was set at 5%, and all tests were 2-sided.

## Results

### Dogs Characteristics

Sixty-one dogs were included in the analysis. There were 13 (21%) mixed breed dogs, 9 (15%) Rottweilers, 4 (7%) Dobermann pinscher, 3 (5%) each of Beagle, Bernese Mountain dog, Border collie, and German shepherd, 2 (3%) each of West Highland white terrier, Golden retriever, Fox terrier, English setter, and Chow Chow, and 1 (2%) each of American Staffordshire terrier, Belgian shepherd, Breton, bull terrier, dachshund, Dalmatian, dogo argentino, English bulldog, Irish setter, Labrador retriever, Pitbull, Poodle, and Vizsla.

There were 29 (48%) females, of which 22 spayed, and 32 (52%) males, of which 7 neutered. Median age was 7 years (range, 3–15), and median weight was 27 kg (range, 5–60).

At diagnosis, 9 (15%) and 11 (18%) dogs were anemic and thrombocytopenic, respectively. Twenty-two dogs (36%) had increased LDH activity, and 16 (26%) had BM infiltration >3%. Four (7%) dogs had stage III disease, 16 (26%) had stage IV disease, and 41 (67%) had stage V disease. Seventeen (28%) dogs were symptomatic at diagnosis, and 14 (23%) dogs received at least one dose of glucocorticoids before admission. Eight (13%) dogs had extranodal involvement of lung (*n* = 6) or skin (*n* = 2).

Eighteen dogs (30%) received anesthesia during their follow-up. All received methadone (*n* = 15; 83%) or buthorphanol (*n* = 3; 17%) for analgesia, propofol for anesthesia induction, and isoflurane (*n* = 16; 89%) or sevoflurane (*n* = 2; 11%) for anesthesia maintenance. Reasons for the dogs to be anesthetized were lymphadenectomy for diagnostic purposes (*n* = 13), computed tomography (*n* = 2), plasma cell tumor excision (*n* = 1), colonic leiomyosarcoma excision (*n* = 1), and endoscopic foreign body removal (*n* = 1). Median anesthesia duration was 30 mins (range, 20–120). No complications related to anesthesia itself or to the surgical procedure, if performed, were reported.

### Outcome

Overall median TTR was 329 days (95% CI, 281–377). In particular, 48 (79%) dogs relapsed during the study period, while 13 (21%) dogs were still in CR at data analysis closure after a median follow-up time of 449 days (range, 270–1,675). On univariable analysis (Table 1), the presence of symptoms at admission (substage b) was the only variable significantly associated with an increased risk of relapse (HR 2.13; 95% CI, 1.16–3.91, *P* = 0.015).

**Table 1:** Results of Cox proportional hazards regression analysis: univariable association of baseline characteristics with lymphoma relapse.

**Baseline characteristic**	**Univariate analysis**		
	**Hazard ratio**	**95% CI**	***P***
Anemia	1.77	0.85–3.70	0.126
Thrombocytopenia	1.08	0.52–2.24	0.839
Increased LDH	1.29	0.72–2.31	0.390
WHO stage V	1.30	0.69–2.45	0.415
Substage b	2.13	1.16–3.91	0.015^*^
BM infiltration >3%	1.47	0.82–2.64	0.192
Extranodal involvement	1.94	0.86–4.38	0.113
Pre-treatment w/ steroids	1.30	0.66–2.57	0.448

* *Significant at the 5% level. CI, confidence interval; LDH, serum lactate dehydrogenase; WHO, World Health Organization; BM, bone marrow*.

**Figure 1:**
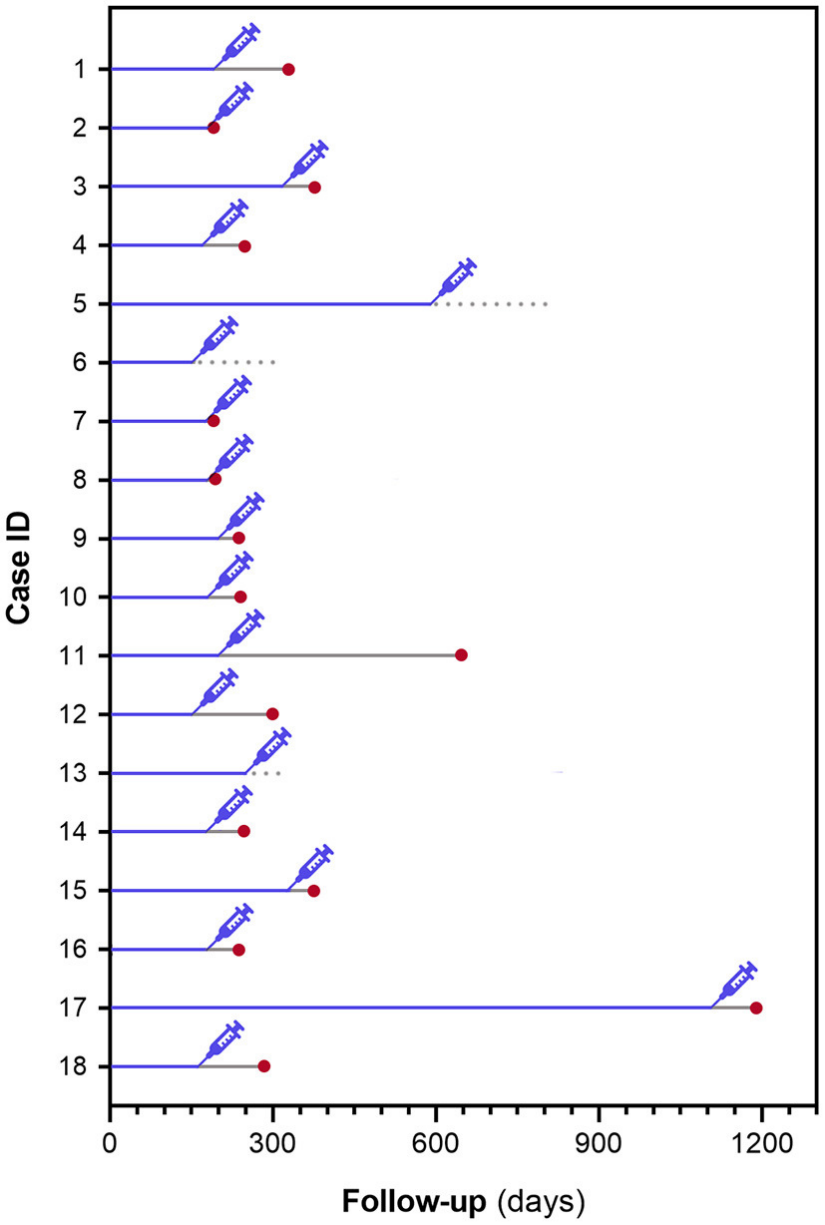
Schematical representation of time from DLBCL diagnosis to anesthesia and time from anesthesia to lymphoma relapse in 18 dogs undergoing an anesthetic procedure.

For the 18 dogs undergoing anesthesia, median time from DLBCL diagnosis to anesthesia was 179 days (range, 148–1, 111; Figure 1). Among them, 15 (83%) relapsed after a median of 73 days (range, 9–449), while 3 dogs did not relapse after 53, 153, and 208 days from anesthesia.

Distribution of variables in cases and matched controls obtained via incidence density sampling is presented in Table 2. According to the logistic conditional regression analysis (Table 3), the relative risk of lymphoma relapse for dogs undergoing anesthesia during their follow-up was significantly higher compared with dogs not undergoing anesthesia (OR 3.44, 95% CI, 1.37–8.65; *P* = 0.009). When the model was adjusted for variables that showed to be differently distributed across the two exposure groups with a significance level of *P* ≤ 0.20 (i.e., anemia, pre-treatment with steroids), the relative risk for relapse remained significantly higher for dogs undergoing anesthesia (OR 3.09; 95% CI, 1.20–7.94; *P* = 0.019).

**Table 2:** Distribution of baseline characteristics in cases and matched controls obtained *via* incidence density sampling.

**Baseline characteristic**	**Cases**	**Controls**	***P***
	**(*n* = 48)**	**(*n* = 96)**	
Anemia	9 (19%)	9 (9%)	0.109
Thrombocytopenia	9 (19%)	14 (15%)	0.520
Increased LDH	19 (40%)	33 (34%)	0.540
WHO stage V	34 (71%)	63 (66%)	0.530
Substage b	16 (33%)	23 (24%)	0.233
BM infiltration	19 (40%)	32 (33%)	0.460
Extra-nodal involvement	7 (15%)	8 (8%)	0.247
Pre-treatment with steroids	11 (23%)	13 (14%)	0.155

**Table 3:** Results of conditional logistic regression analysis: odds ratios of disease progression associated with exposure to anesthesia.

	Odds ratio^*^	***P***	**95% CI**	
			**Lower**	**Upper**
Crude	3.44	0.009^*^	1.37	8.65
Adjusted^†^	3.09^*^	0.019	1.20	7.94

* *Unbalanced estimate of the relative risk*.

† *Adjusted for anemia and pre-treatment with steroids*.

*Significant at the 5% level.

## Discussion

Immune cells are critical components of the tumor microenvironment, and it has become evident that the immune system plays a complex and multi-faceted role in cancer, by affecting tumor promotion and prevention. The evidence from experimental laboratory and human clinical studies supports that anesthetics can modulate the immune system, thereby influencing the metastatic behavior of cancer cells (5–12).

In the present study, more than 80% of dogs with DLBCL undergoing anesthesia with opioids, propofol, and isoflurane/sevoflurane while in CR relapsed on average within 73 days from the procedure, with a risk of relapse more than 3 times higher compared with dogs not receiving anesthesia.

This finding is not surprising, as a possible immunosuppressive effect of several anesthetic drugs is well-documented in the literature. It is suggested that volatile anesthetics such as sevoflurane and isoflurane may favor neoplastic cell spread by inhibiting NK cell cytotoxicity, inducing T-lymphocyte apoptosis, and enhancing angiogenesis through hypoxia inducible factor-1α activity (6, 22, 23). In contrast, TIVA is suggested to have anti-inflammatory properties, and to oppose tumor spread by promoting the activation of T-helper cells, decreasing matrix metalloproteinases, and suppressing NK cell activity to a lesser extent than inhalant drugs (6–8, 24).

From a clinical point of view, few retrospective studies in people have proposed a possible association between the type of anesthetic protocol and outcome, suggesting that loco-regional anesthesia and TIVA might be preferable for the surgical removal of some malignant tumors, including breast and colorectal cancer (10–12).

In companion animals, only 2 studies have investigated the possible effects of anesthesia on the immune system, and documented significant decreases of CD3^+^ lymphocytes, CD4^+^ lymphocytes, CD8^+^ lymphocytes, and NK cytotoxic activities in healthy dogs undergoing anesthesia with isoflurane (13, 14).

The current study is the first evaluating a possible *in vivo* effect of anesthesia on the outcome of cancer-bearing dogs. All patients in our series received opioids, isoflurane or sevoflurane, and propofol, with the latter used only for induction. As no dog received TIVA, it was presumed that the potential protective effect of propofol might have been outclassed by the negative effects mediated by opioids and inhalant drugs. Unfortunately, we did not have any immune monitoring data for the dogs in this study, which would have been useful to substantiate the observed association between anesthesia and lymphoma relapse.

Besides surgery, chemotherapy, and radiotherapy, immunotherapy has become the fourth strategy against cancer, aiming at activating the immune system of the patient to recognize and fight cancer cells otherwise recognized as self. All dogs in our series received CHOP-based chemotherapy with the incorporation of APAVAC vaccine, an immunotherapeutic product which has shown to improve outcome in dogs with DLBCL (17, 18). The rationale behind its efficacy is attributable to the mounting of a cell-mediated immune response against patient's tumor-associated antigens. Considering that anesthesia might decrease, although temporarily, T-lymphocytes and NK cells activity, the newly acquired cell-mediated immunity may have been impaired, thereby promoting relapse. It is unknown whether the observed association would have still emerged if dogs had been treated with chemotherapy only.

In human oncology, anesthesia is almost always part of the therapeutic protocol only, being linked to surgery for the excision of primary or secondary lesions. In dogs, anesthesia is also frequently performed for diagnostic and staging procedures, either when organ samples need to be taken or for the management of aggressive or exceedingly fearful subjects, and maintenance for longer procedures is typically obtained with volatile anesthetics. With the demonstration of a possible negative effect on outcome, clinicians may be called to carefully evaluate pros and cons of all diagnostic investigations requiring anesthesia (e.g., BM sampling, ultrasound-guided fine needle aspiration of abdominal organs). Moreover, as TIVA is considered a safe alternative to anesthetic protocols including volatile drugs, additional studies comparing the effects of propofol and inhalant drugs on small animals' immune system are warranted.

Anesthesia is a time-dependent variable in statistical analysis, as its need occurs randomly during the disease course, thus assessing its possible role as a prognostic factor is challenging in a cohort study. Making a classical comparison of the TTR of anesthetized dogs with that of dogs not undergoing anesthesia might leave space for a time-window bias resulting from the use of time-windows of different lengths between cases and controls to define time-dependent exposures (21). In order to obviate this, we used a time-matched nested case-control design, by which the relative risk of lymphoma relapse related to anesthesia was assessed, and this provided significant effect sizes both in univariable and multivariable analyses.

Further limits of this study are due to its retrospective nature, not allowing for the immune monitoring of patients, and to the limited number of cases. However, the included population was homogeneous in terms of lymphoma histotype, initial staging, treatment, and end-staging, and well-balanced for known prognostic variables when evaluating a possible interference of anesthesia on relapse. Moreover, the only three dogs receiving anesthesia that did not relapse in the present study had a limited follow-up time, therefore possibly underestimating the impact of anesthesia on recurrence.

It must be acknowledged that factors other than anesthesia might contribute to an immunosuppressive status, ultimately promoting cancer progression, and these may include surgery and perioperative stress (4). Indeed, most of the dogs in this study underwent a concurrent surgical procedure, and it is not possible to reliably assess which factor has mainly contributed to the observed disease relapse. Moreover, anesthesia may have just forestalled a soon-to-happen relapse, and whether this association has an influence on survival time has still to be determined.

In conclusion, our findings suggest that anesthesia may promote relapse in dogs with DLBCL treated with chemo-immunotherapy. Considering the high frequency of anesthetic procedures required in veterinary oncology diagnostic and therapeutic protocols, it is of utmost interest to characterize the effects of single anesthetic agents on the immune system and verify whether the same conclusion can be drawn for dogs treated with chemotherapy only.

## Data Availability Statement

The original contributions presented in the study are included in the article/Supplementary Material, further inquiries can be directed to the corresponding author.

## Ethics Statement

Ethical review and approval was not required for the animal study because retrospective study—nothing but the gold standard was performed on owned dogs. Written informed consent was obtained from the owners for the participation of their animals in this study.

## Author Contributions

LM conceived the presented idea and planned the study. LM, EF, DG, SC, LA, AM, SZ, VC, and EL collected the data. SS and JL performed the statistical analysis and the data interpretation. LM, EF, and SS drafted the manuscript and prepared the tables and the figures. LM, EF, SS, JL, SC, LA, and SZ revised the manuscript. All authors discussed the results and approved the final version of the manuscript.

## Conflict of Interest

The authors declare that the research was conducted in the absence of any commercial or financial relationships that could be construed as a potential conflict of interest.

## Publisher's Note

All claims expressed in this article are solely those of the authors and do not necessarily represent those of their affiliated organizations, or those of the publisher, the editors and the reviewers. Any product that may be evaluated in this article, or claim that may be made by its manufacturer, is not guaranteed or endorsed by the publisher.
