# Supplementary material for: Sleeping Beauty: Anesthesia May Promote Relapse in Dogs With Diffuse Large B-Cell Lymphoma in Complete Remission After Chemo-Immunotherapy
Source: Front Vet Sci. 2021 Nov 22;8:760603. doi: 10.3389/fvets.2021.760603 (PMC8645585; doi:10.3389/fvets.2021.760603)
Supplement: Supplementary file 1 [file Data_Sheet_1.docx]

Supplementary Material

**
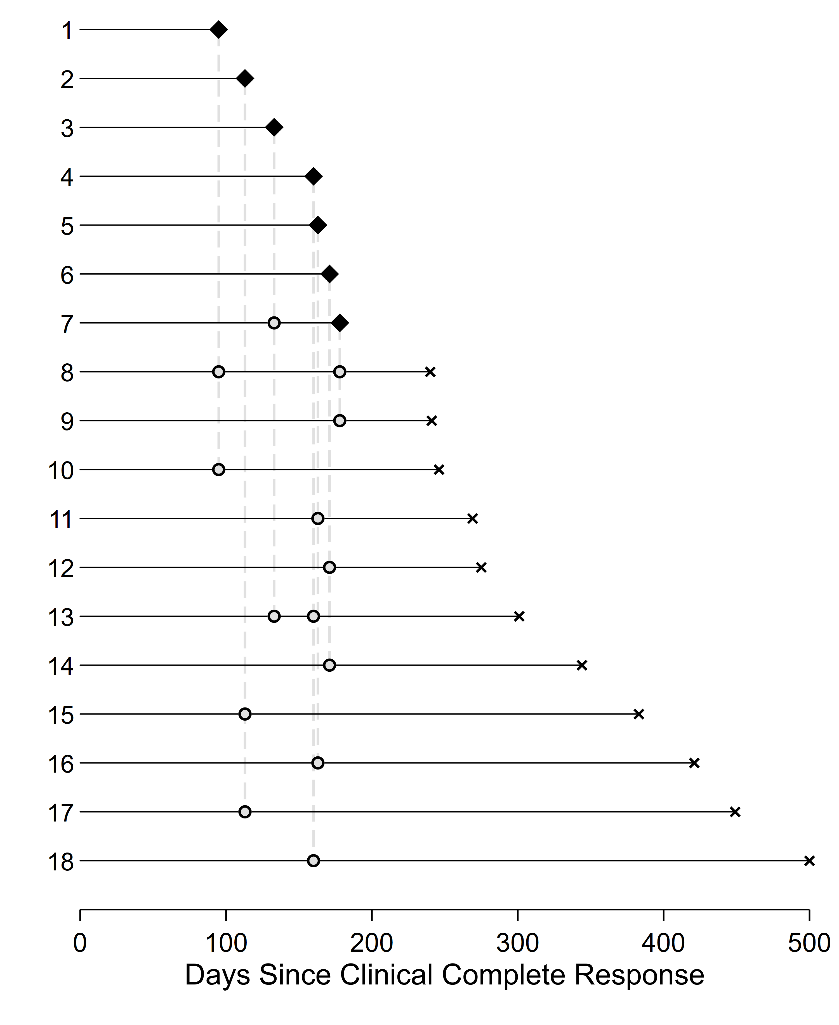
**

**Supplementary Figure 1.** Illustrative example of the nested case-control design used in this study. There are 2 controls (*gray* *circles*) for every case (*black* *diamond*). Follow-up periods for the 18 members of the cohort (*horizontal black lines*) begin at the zero-time on the time axis, which is the date of clinical complete response. No events before the end of the individual follow-up are treated as censored events (*x signs*). Controls are randomly selected from the risk sets (*vertical dashed lines*) of each case. A future case may be selected as a control for a prior case (patient #7), and a subject may be selected as a control for 2 different cases (patients #8 and #13). Exposure to anesthesia is investigated between the cohort entry and the matching date.
